# Translatable plasma and CSF biomarkers for use in mouse models of Huntington’s disease

**DOI:** 10.1093/braincomms/fcae030

**Published:** 2024-02-07

**Authors:** Marie K Bondulich, Jemima Phillips, María Cañibano-Pico, Iulia M Nita, Lauren M Byrne, Edward J Wild, Gillian P Bates

**Affiliations:** Huntington’s Disease Centre, Department of Neurodegenerative Disease and UK Dementia Research Institute at UCL, Queen Square Institute of Neurology, UCL, London WC1N 3BG, UK; Huntington’s Disease Centre, Department of Neurodegenerative Disease and UK Dementia Research Institute at UCL, Queen Square Institute of Neurology, UCL, London WC1N 3BG, UK; Huntington’s Disease Centre, Department of Neurodegenerative Disease and UK Dementia Research Institute at UCL, Queen Square Institute of Neurology, UCL, London WC1N 3BG, UK; Huntington’s Disease Centre, Department of Neurodegenerative Disease and UK Dementia Research Institute at UCL, Queen Square Institute of Neurology, UCL, London WC1N 3BG, UK; Huntington’s Disease Centre, Department of Neurodegenerative Disease and UK Dementia Research Institute at UCL, Queen Square Institute of Neurology, UCL, London WC1N 3BG, UK; Huntington’s Disease Centre, Department of Neurodegenerative Disease and UK Dementia Research Institute at UCL, Queen Square Institute of Neurology, UCL, London WC1N 3BG, UK; Huntington’s Disease Centre, Department of Neurodegenerative Disease and UK Dementia Research Institute at UCL, Queen Square Institute of Neurology, UCL, London WC1N 3BG, UK

**Keywords:** Huntington’s disease, mouse plasma and CSF biomarkers, neurofilament light chain, Tau, YKL-40 and BRP-39

## Abstract

Huntington’s disease is an inherited neurodegenerative disorder for which a wide range of disease-modifying therapies are in development and the availability of biomarkers to monitor treatment response is essential for the success of clinical trials. Baseline levels of neurofilament light chain in CSF and plasma have been shown to be effective in predicting clinical disease status, subsequent clinical progression and brain atrophy. The identification of further sensitive prognostic fluid biomarkers is an active research area, and total-Tau and *YKL-40* levels have been shown to be increased in CSF from Huntington’s disease mutation carriers. The use of readouts with clinical utility in the preclinical assessment of potential therapeutics should aid in the translation of new treatments. Here, we set out to determine how the concentrations of these three proteins change in plasma and CSF with disease progression in representative, well-established mouse models of Huntington’s disease. Plasma and CSF were collected throughout disease progression from R6/2 transgenic mice with CAG repeats of 200 or 90 codons (R6/2:Q200 and R6/2:Q90), zQ175 knock-in mice and YAC128 transgenic mice, along with their respective wild-type littermates. Neurofilament light chain and total-Tau concentrations were quantified in CSF and plasma using ultrasensitive single-molecule array (Quanterix) assays, and a novel Quanterix assay was developed for breast regression protein 39 (mouse homologue of YKL-40) and used to quantify breast regression protein 39 levels in plasma. CSF levels of neurofilament light chain and plasma levels of neurofilament light chain and breast regression protein 39 increased in wild-type biofluids with age, whereas total-Tau remained constant. Neurofilament light chain and breast regression protein 39 were elevated in the plasma and CSF from Huntington’s disease mouse models, as compared with wild-type littermates, at presymptomatic stages, whereas total-Tau was only increased at the latest disease stages analysed. Levels of biomarkers that had been measured in the same CSF or plasma samples taken at the latest stages of disease were correlated. The demonstration that breast regression protein 39 constitutes a robust plasma biomarker in Huntington’s disease mouse models supports the further investigation of *YKL-40* as a CSF biomarker for Huntington’s disease mutation carriers. Neurofilament light chain and Tau are considered markers of neuronal damage, and breast regression protein 39 is a marker of inflammation; the similarities and differences in the levels of these proteins between mouse models may provide future insights into their underlying pathology. These data will facilitate the use of fluid biomarkers in the preclinical assessment of therapeutic agents for Huntington’s disease, providing readouts with direct relevance to clinical trials.

## Teaser

Bondulich *et al*. show that levels of NEFL, total-Tau and BRP-39 in plasma and CSF will provide translatable readouts for preclinical studies in Huntington’s disease mouse models. Further analysis in mice may shed light on the underlying pathologies leading to elevated biomarker levels.

## Graphical Abstract

**Graphical Abstract:**
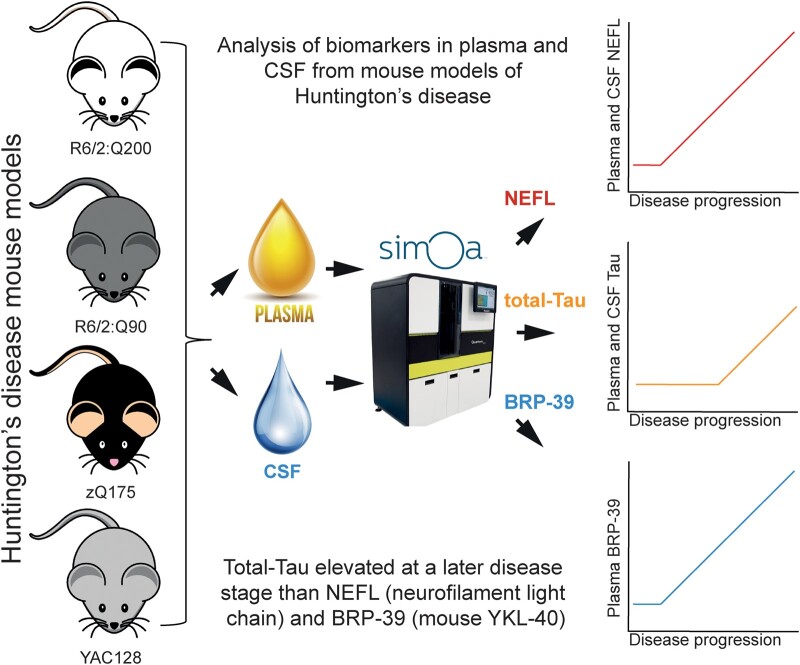

## Introduction

Huntington’s disease is a devastating inherited neurodegenerative disorder characterized by motor, cognitive and psychiatric dysfunction.^1^ It is caused by a CAG triplet repeat expansion within exon 1 of the huntingtin gene (*HTT*) resulting in an abnormally long polyglutamine tract (polyQ) within the huntingtin protein (HTT).^2^ Individuals with 35 CAGs or less are unaffected, those with 40 or more will develop the disease within a normal lifespan, and CAG repeats of ∼65 or more will cause disease onset in childhood or adolescence.^3^ The CAG repeats are unstable on transmission, with expansions generally occurring upon inheritance from a male.^4^ Expanded repeats are also somatically unstable, with expansions amounting to 100 s of CAGs occurring in specific brain regions.^5-8^ Although the CAG repeat length in blood correlates with the age of onset and progression of the disease, genetic and environmental factors are also known to contribute.^9,10^ Recently, genome-wide association studies identified DNA mismatch repair genes as genetic modifiers.^11-14^ This has led to the widely held hypothesis that somatic CAG repeat expansion is the first step in the Huntington’s disease pathogenesis, as nullizygosity for some of these genes was known to prevent somatic CAG repeat instability in Huntington’s disease mouse models.^15,16^ The alternative processing of the *HTT* pre-mRNA, to generate the *HTT1a* transcript, that encodes the highly aggregation-prone and pathogenic HTTexon1 protein increases with increasing CAG repeat length and is a candidate for the second step in molecular pathogenesis of this disease.^17,18^ Neuropathologically, the disease is characterized by intranuclear and extranuclear inclusion bodies^19,20^ and neuronal cell death in the striatum, cortex and other brain regions.^21,22^

There are currently no approved disease-modifying treatments for Huntington’s disease, but advances in our understanding of its pathogenesis have meant that there are many potential disease-modifying strategies in preclinical and clinical development.^23^ These include approaches to lower the levels of HTT through the administration of antisense oligonucleotides, RNA interference, zinc finger proteins and small molecules.^24^ The identification of DNA repair genes as genetic modifiers has induced wide-ranging strategies to target these genes and decrease somatic CAG repeat instability in the brain.^23^ Irrespective of the approach, it will be important to conduct clinical trials at an early timepoint in the disease, before the onset of observable clinical signs. To facilitate this, a new evidence-based integrated clinical staging system has been developed that incorporates all available data including biomarkers.^25^ Within the staging system, imaging biomarkers were chosen to define the process of neurodegeneration and the boundary between stages 0 and 1^25^; however, fluid biomarkers may also aid in the interpretation of this transition.^26^ Increased levels of neurofilament light chain (NEFL) have been detected in the plasma and CSF of Huntington’s disease patients before the onset of symptoms, and baseline levels have been shown to predict clinical disease status, subsequent clinical progression and brain atrophy.^27,28^ NEFL is currently the strongest monitoring and prognostic fluid biomarker for Huntington’s disease.^28^ Other fluid biomarkers that have been reported to correlate with clinical phenotypes of Huntington’s disease include the HTT protein^29^ total-Tau^30^ and YKL-40.^31^

Mouse models are frequently used for the target validation of therapeutic strategies and the preclinical testing of potential therapeutic agents, the predictive value of which should be increased if the outcome measures are directly translatable to the clinic. To date, serum and CSF NEFL levels have only been reported in R6/2 mice with CAG expansions of 273–285, in which they were shown to increase with disease progression.^32^ To generate more comprehensive data sets, we performed longitudinal measures of the plasma and CSF levels of NEFL, total-Tau and breast regression protein 39 (BRP-39) (mouse YKL-40) in mouse models of Huntington’s disease. We selected three widely used models that are representative of the transgenic and knock-in lines that are available.^33^ R6/2 mice are transgenic for the 5ʹ end of the human *HTT* gene that encodes the HTTexon1 protein at endogenous *Htt* levels and are good models of HTT aggregation and its downstream consequences.^34^ We selected two R6/2 lines with CAG repeat expansions of 200 (R6/2:Q200) and of 90 (R6/2:90).^35^ The zQ175 knock-in mice were created by replacing mouse *Htt* exon 1 with a mutated version of human *HTT* exon 1 (∼190 CAGs); they develop phenotypes at younger ages than other knock-in models^36,37^ and have been used to evaluate HTT lowering therapies.^38^ YAC128 mice expresses the human *HTT* locus as a transgene^39,40^ and play a pivotal role in preclinical screening for therapeutics specifically targeting the human *HTT* sequence.^41^ All three biomarkers were assessed in plasma, and NEFL and total-Tau were also measured in CSF. Plasma NEFL and BRP-39, and CSF NEFL, levels were raised at early- to mid-stage disease in all the Huntington’s disease mouse models, whereas total-Tau was only elevated at later stages of disease in both plasma and CSF. All three proteins have the potential to provide valuable translatable biomarkers for Huntington’s disease preclinical studies.

## Materials and methods

### Ethics statement

All procedures were performed in accordance with the Animals (Scientific Procedures) Act 1986 and were approved by the UCL Ethical Review Panel.

### Mouse breeding and maintenance

Hemizygous R6/2:Q200 mice were bred by backcrossing R6/2 males to (C57BL/6JOlaHsd × CBA/CaOlaHsd)F1 females (B6CBAF1/OlaHsd; Envigo, Netherlands). YAC128 and R6/2:Q90 mice were bred in-house by backcrossing to C57BL/6J (Charles River). zQ175 mice on a C57BL/6J background were imported from the CHDI Foundation colony at the Jackson Laboratory (Bar Harbor, Maine). Mouse breeding and maintenance was performed as previously described.^42^ Mice for each colony were group-housed depending on gender, and genotypes were mixed within cages. All animals were kept in individually ventilated cages containing Aspen Chips 4 Premium bedding (Datesand) with environmental enrichment in the form of chew sticks and a play tunnel (Datesand). All mice had *ad libitum* access to water and chow (Teklad global 18% protein diet, Envigo, the Netherlands). The temperature was automatically regulated at 21°C ± 1°C and animals were kept on a 12 h light/dark cycle. The animal facility was barrier-maintained, and quarterly non-sacrificial Federation of European Laboratory Animal Science Associations (FELASA) screens found no evidence of pathogens. Mice in the R6/2 colonies developed neurological phenotypes over the time scale of the study. They were sacrificed for biofluid collection before reaching a humane endpoint as judged by weight gain and a disease-stage rating scale.

### Genotyping and CAG repeat sizing

DNA was prepared from ear biopsy, and mice were genotyped as previously described.^43^ All primers were from Invitrogen. For CAG repeat sizing, 50 ng DNA was amplified using HD3F FAM 5ʹ-CCTTCGAGTCCCTCAAGTCCTT-3ʹ and HD5 5ʹ-CGGCTGAGGCAGCAGCGGCTGT-3ʹ primers with Amplitaq Gold and GC enhancer (Thermo Fisher Scientific). Cycling conditions were 95°C for 10 min; 35 cycles of 95°C for 30 s, 65°C for 30 s and 72°C for 90 s; and 72°C for 10 min. Samples were run on an ABI 3730xl Genetic Analyser with MapMarker ROX 1000 internal size standards and analysed using GeneMapper v5 software (Thermo Fisher Scientific). The mean CAG repeat size for R6/2:Q90 mice was 90.55 ± 1.79 (±SD), for R6/2:Q200 mice was 210.24 ± 7.2 and for zQ175 mice was 191.8 ± 1.8. The polyQ repeat of 125 glutamines in YAC128 mice is encoded by (CAG)_23_(CAA)_3_CAGCAA(CAG)_80_(CAA)_3_CAGCAA(CAG)_10_CAACAG which is stable on germline transmission.^44^

### Plasma and CSF collection

Blood was collected into ethylenediaminetetraacetic acid tubes via terminal cardiac puncture from R6/2:Q200 and wild-type littermates at 4, 8 and 12 weeks of age; from R6/2:Q90 and wild-type littermates at 4, 14 and 24 weeks of age; from zQ175 and wild-type littermates at 2, 6 and 12 months of age; and from YAC128 and wild-type littermates at 2, 6 and 12 months of age. Plasma was extracted after centrifugation at ∼2500 × *g* for 15 min and stored at −80°C. For CSF collection, terminally anaesthetized mice were placed in a stereotaxic frame, and the skin and muscle tissue were dissected to expose the dura mater. The arachnoid membrane covering the cisterna magna was then punctured using a mouth-controlled glass capillary micropipette. CSF was collected from zQ175 and wild-type littermates at 6 and 12 months of age, R6/2:Q200 and wild-type littermates at 12 weeks of age, R6/2:Q90 and wild-type littermates at 16 and 24 weeks of age and YAC128 and wild-type littermates at 6 and 12 months of age and stored at −80°C.

### NEFL and total-Tau measurements

Mouse plasma and CSF NEFL concentration was quantified using commercially available NF-light™ Advantage Kit (103186, Quanterix). Total-Tau concentrations were quantified using the mouse total-Tau assay (102209, Quanterix) which detects the mid-protein epitope of the Tau protein. Plasma samples were diluted 1:40 and CSF samples 1:100 in the sample buffer provided in the kits as per manufacturer instructions. Standards and samples were run in technical duplicates for single-molecule array (SIMOA) immunoassay and run on the HD-X analyzer using the two-step assay as per manufacturer’s instructions (Quanterix). For NEFL, the lower limit of quantification (LLOQ) was 0.174 pg/mL, and the limit of detection (LOD) was 0.038 pg/mL. For total-Tau, the LLOQ was 0.823 pg/mL, and the limit of detection was 0.428 pg/mL. The intra-assay coefficients of variability of replicates were automatically calculated by the instrument to assess the repeatability of the test and expressed as percentages.

### Simoa assay for quantification of BRP-39

We developed an ultrasensitive digital immunoassay using the Quanterix Homebrew Assay Development Kit to create a rapid prototype single-molecule array (Simoa®) assay for the purpose of measuring BRP-39 in mouse plasma. Antibody bead conjugation and biotinylation were performed as recommended by Quanterix’s Homebrew Assay Development guide. In brief, anti-BRP-39 capture beads were prepared by covalent coupling of 0.2 mg/mL anti-YKL-40/CHI3L1 capture antibody (antibody pair, ab244254) to 0.3 mL of carboxyl paramagnetic microbeads and 0.3 mg/mL 1-ethyl-3-(3-dimethylaminopropyl) carbodiimide with conjugation performed at 2–8°C. Mouse anti-YKL-40/CHI3L1 detector antibody (antibody pair, ab244254) was biotinylated according to the supplier protocol. For each biotinylation, 100 μg of antibody was used at 1 mg/mL and a 40:1 ratio of NHS-PEG4-biotin to antibody.

Assay optimization was performed by testing a two-step versus a three-step sandwich immunoassay on the HD-X analyzer (Quanterix). Detector antibody concentrations were from 0.3 to 1.5 μg/mL, and streptavidin-β-D-galactosidase concentrations were from 50 to 150 pM. Multiple assay combinations were run in parallel to enable selection of optimal conditions. In brief, the first step combines capture beads, sample and biotinylated antibody to form a sandwich complex; after incubation and wash steps, streptavidin beta-galactosidase was added to cuvettes to label the complex. After the fully labelled complex had formed, beads were washed and transferred to a Simoa disc where the beads were isolated in femtoliter-sized microwells, sealed in the presence of substrate resorufin β-d-galactopyranoside and analysed for the presence of enzyme label. A BRP-39 standard curve was prepared from recombinant BRP-39 (ab238262) at 17 000 pg/mL, which was serially diluted in sample diluent (ab221827) to create a nine-point standard curve and blank control. High (2500 pg/mL), middle (160 pg/mL) and low (10.2 pg/mL) quality control samples were prepared independently for each assay from a 17 000 pg/mL stock of BRP-39. Calibration curves were prepared, and the LOD and LLOQ were determined. The LOD was 3.5 ng/mL and established by adding three times the standard deviation of the background to the background signal. LLOQ was 5.8 pg/mL and was determined as the BRP-39 concentration at which a coefficient of variation of 20% was observed. Mouse plasma (R6/2:Q200 and wild type) samples were thawed on ice and centrifuged at 14 000 × *g* for 15 min at room temperature and then diluted to 1:40 in sample diluent (ab221827). This dilution had been previously determined by dilutional linearity and spike recovery, whereby samples from pooled wild-type and R6/2:Q200 plasma samples were either spiked with recombinant protein or left unspiked and then serially diluted. Comparison of spiked to unspiked samples showed that the optimum percentage recovery had been achieved (80–120%). Therefore, the optimum dilution was determined to be between 1:40 and 1:80.

### Statistical analysis

Data were screened for outliers using ROUT or Grubb’s test (GraphPad Prism v7), and any outliers were removed before between-group comparisons. All data sets were tested for a normal Gaussian distribution (Shapiro–Wilk, Prism v7). Statistical analysis was performed with SPSS (v26) for two-way ANOVA or general linear model (GLM) ANOVA, with Bonferroni *post hoc* tests as indicated. Graphs were prepared using GraphPad Prism (v7) and R. *P* values < 0.05 were considered statistically significant. The association between parameters was examined for zQ175, R6/2:Q200, R6/2:Q90 and YAC128 plasma and CSF at late-stage disease using Pearson’s correlation analysis, and graphs were plotted with curves indicating 95% confidence interval from a linear regression line of best fit through the data set.

## Results

Plasma was collected from the Huntington’s disease mouse models at ages corresponding to different stages of disease progression. R6/2:Q200 mice were selected at 4, 8 and 12 weeks of age and R6/2:Q90 mice at 4, 14 and 24 weeks of age. In both cases, these ages correspond to presymptomatic, mid-stage and late-stage disease.^35^ For the zQ175 and YAC128 lines, plasma was collected at 2, 6 and 12 months of age. These ages were chosen, as they are likely to span the duration of a preclinical therapeutic trial in either of these models but correspond to earlier stages of disease than those for the R6/2 lines. HTT aggregation and transcriptional dysregulation signatures are pronounced for the zQ175 knock-in mice at 6 months of age,^37,45^ but behavioural changes develop more slowly and even at 12 months can be relatively subtle in heterozygous mice.^36,46^ Disease phenotypes progress more slowly for YAC128 than for zQ175 mice. HTT aggregation in the brain can be detected by 3 months of age,^42^ but even at 12 months, there is considerable variation between labs in the level of behavioural impairment that can be detected.^47,48^

**Figure 1:**
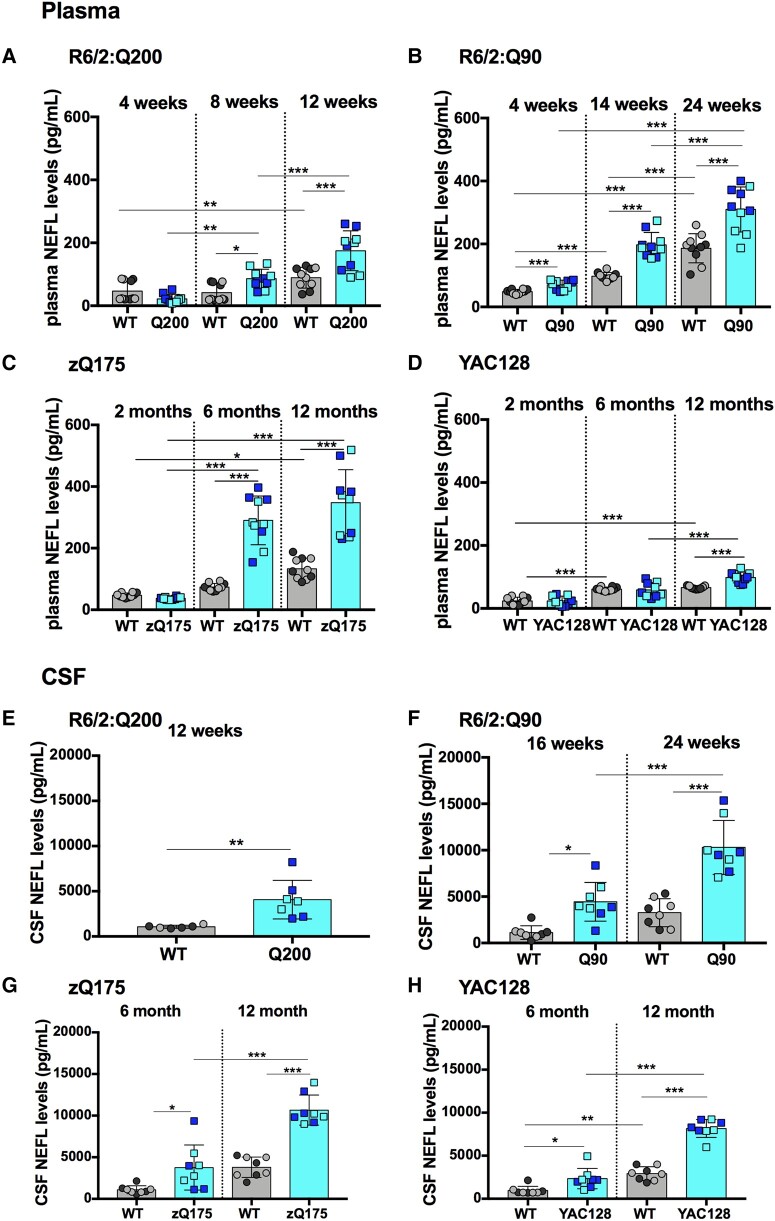
**NEFL plasma and CSF levels are elevated with disease progression in R6/2Q200, R6/2Q90, zQ175 and YAC128 mice.** (**A**–**D**) NEFL plasma levels measured by Simoa at three stages of disease for R6/2:Q200, R6/2:Q90, zQ175 and YAC128 as compared with wild-type littermates. (**A**) R6/2:Q200 showed no change at 4 weeks of age, and levels increased at 8 and 12 weeks of age. (**B**) R6/2:Q90 showed an increase at 4, 14 and 24 weeks of age. (**C**) zQ175 showed no change at 2 months of age and an increase at 6 and 12 months of age. (**D**) YAC128 showed an increase at 12 months of age only. In all cases, NEFL levels increased with age in wild-type mice. Number of mice = 5/gender/genotype. Three outliers were removed for the YAC128 group. (**E**–**H**) NEFL CSF levels measured by Simoa comparing late stage of disease for R6/2:Q200 and two stages of disease for R6/2:Q90, zQ175 and YAC128 as compared with wild-type littermates. (**E**) R6/2:Q200 showed an increase at 12 weeks of age. (**F**) R6/2:Q90 showed an increase at 16 and 24 weeks of age. (**G**) zQ175 showed an increase at 6 and 12 months of age. (**H**) YAC128 showed an increase at 6 and 12 months of age. NEFL levels only increased with age in wild-type mice from the YAC128 colony. Number of mice = 4/gender/genotype. One outlier was removed for the R6/2:Q200 group. Statistical analysis was two-way ANOVA with Bonferroni *post hoc* correction. Error bars = SEM. *P ≤ 0.05, **P ≤ 0.01, ***P ≤ 0.001. The test statistic, degrees of freedom and *P* values for the ANOVA are provided in Supplementary Tables 2 and 3. WT, wild type. Dark circles and squares, females. Lighter circles and squares, males.

**Figure 2:**
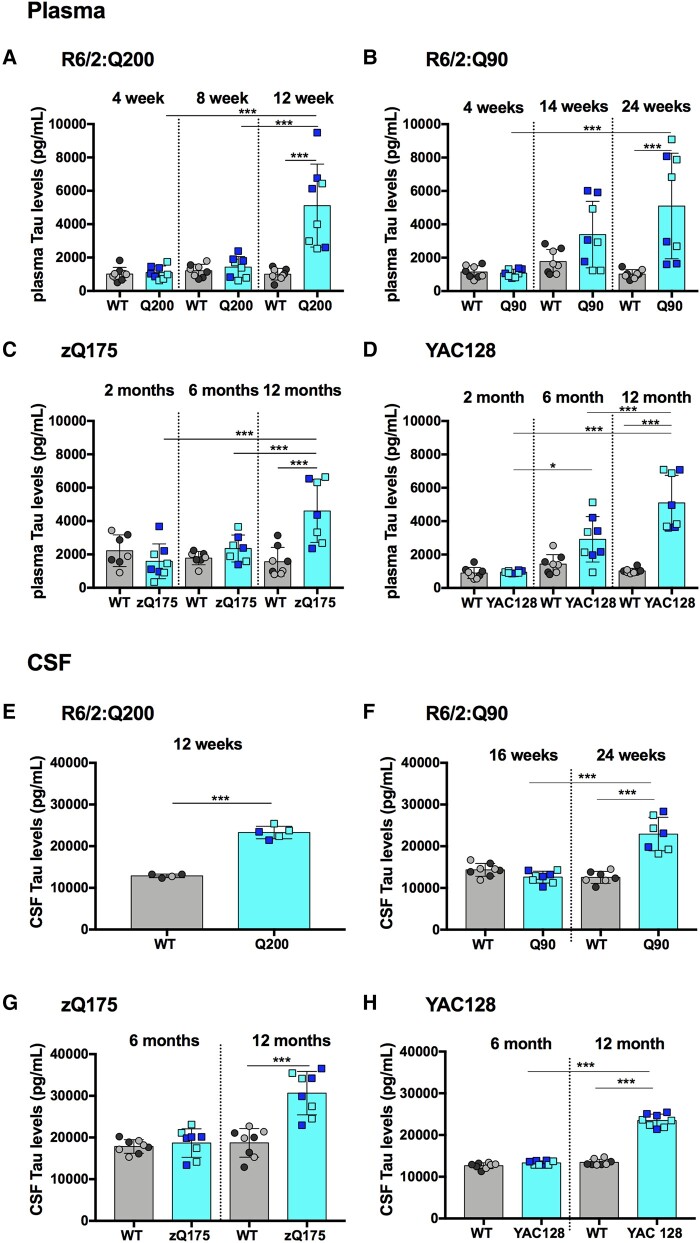
**Total-Tau plasma and CSF levels are elevated only at late stage in R6/2Q200, R6/2Q90, zQ175 and YAC128 mice.** (**A**–**D**) Total-Tau plasma levels measured by Simoa at three stages of disease for R6/2:Q200, R6/2:Q90, zQ175 and YAC128 as compared with wild-type littermates. (**A**) R6/2:Q200 showed no change at 4 and 8 weeks of age and an increase at 12 weeks. (**B**) R6/2:Q90 showed no change at 4 and 14 weeks of age and an increase at 24 weeks. (**C**) zQ175 showed no change at 2 and 6 months of age and an increase at 12 months. (**D**) YAC128 showed no change at 2 and 6 months of age and an increase at 12 months. Number of mice = 4/gender/genotype. Four outliers were removed for zQ175. (**E**–**H**) Total-Tau CSF levels measured by Simoa comparing late-stage disease for R6/2:Q200 and 2 stages of disease for R6/2:Q90, zQ175 and YAC128 as compared with wild-type littermates. (**E**) R6/2:Q200 showed an increase at 12 weeks of age. (**F**) R6/2:Q90 showed no change at 16 weeks of age and an increase at 24 weeks. (**G**) zQ175 showed no change at 6 months of age and an increase at 12 months. (**H**) YAC128 showed no change at 6 months of age and an increase at 12 months. Number of mice = 5 per genotype for R6/2:Q200 and 4/gender/genotype for R6/2:Q90, zQ175 and YAC128. One outlier removed for wild-type mice and two for R6/2:Q200. Statistical analysis was two-way ANOVA with Bonferroni *post hoc* correction. Error bars = SEM. *P ≤ 0.05, **P ≤ 0.01, ***P ≤ 0.001. The test statistic, degrees of freedom and *P* values for the ANOVA are provided in Supplementary Tables 4 and 5. WT, wild type. Dark circles and squares, females. Lighter circles and squares, males.

**Figure 3:**
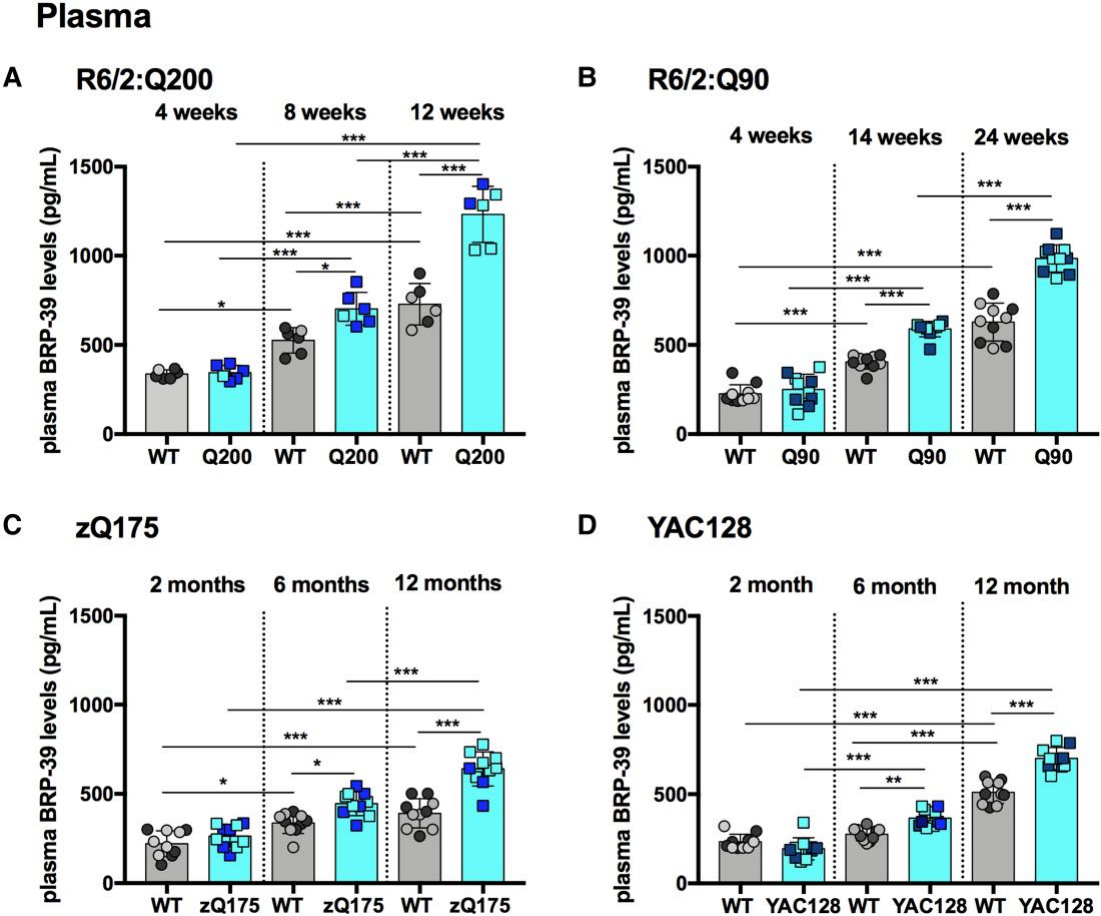
**BRP-39 plasma levels are elevated with disease progression in R6/2:Q200, R6/2:Q90, zQ175 and YAC128 mice.** (**A**–**D**) BRP-39 plasma levels measured by Simoa at three stages of disease for R6/2:Q200, R6/2:Q90, zQ175 and YAC128 as compared with wild-type littermates. (**A**) R6/2:Q200 showed no change at 4 weeks of age and an increase at 8 and 12 weeks. (**B**) R6/2:Q90 showed no change at 4 weeks of age and an increase at 14 and 24 weeks. (**C**) zQ175 showed no change at 2 months of age and an increase at 6 and 12 months. (**D**) YAC128 showed no change at 2 months of age and an increase at 6 and 12 months. In all cases, BRP-39 levels increased with age in wild-type mice. Number of mice = 3/gender/genotype for R6/2:Q200 and 5/gender/genotype for R6/2:Q90, zQ175 and YAC128. Statistical analysis was two-way ANOVA with Bonferroni *post hoc* correction. Error bars = SEM. *P ≤ 0.05, **P ≤ 0.01, ***P ≤ 0.001. The test statistic, degrees of freedom and *P* values for the ANOVA are provided in Supplementary Table 6. WT, wild type. Dark circles and squares, females. Lighter circles and squares, males.

Whilst plasma was collected at all ages, CSF was not collected from mice at 1–2 months of age, as the volume generally retrieved from these young mice was low. CSF was collected from separate cohorts of mice to those used for plasma at the following ages: R6/2:Q200, 12 weeks of age; R6/2;Q90, 16 and 24 weeks; and zQ175 and YAC128 at 6 and 12 months of age. In all cases, an equal number of male and female mice were used, and the data points in Figs. 1–3 are colour-coded to indicate gender. NEFL and total-Tau were measured in plasma and CSF. We designed a homebrew Simoa assay that would allow us to detect BRP-39 in plasma, but unfortunately, it did not have sufficient CSF from one cohort of mice to validate and run the BRP-39 assay.

### NEFL levels were elevated in plasma and CSF with disease progression in R6/2:Q200, R6/2:Q90, zQ175 and YAC128 compared with wild-type mice

NEFL levels were found to increase in wild-type plasma with age. For the two R6/2 cohorts, wild-type levels increased between 4 weeks and 12–14 weeks of age by ∼2-fold (Fig. 1A and B) and then increased again between 14 and 24 weeks of age by a similar amount (Fig. 1B). In the zQ175 and YAC128 cohorts, wild-type NEFL levels increased in a stepwise manner between 2 and 12 months of age (Fig. 1C and D), with higher levels detected in wild-type mice from the zQ175 colony than those from the YAC128 colony at 12 months (Fig. 1C and D).

Increased plasma NEFL levels were detected for all Huntington’s disease mouse lines when compared with wild-type littermates. For R6/2:Q200 mice, plasma NEFL concentrations were elevated by 2-fold at 8 and 12 weeks of age (Fig. 1A). For R6/2:Q90 a significant change of 1.4-fold was observed as early as 4 weeks of age, with 2-fold and 1.7-fold increases being detected at 14 and 24 weeks of age, respectively (Fig. 1B). For zQ175, plasma NEFL concentrations were increased by 3.9-fold and 2.6-fold at 6 and 12 months of age, respectively (Fig. 1C), whereas for YAC128, an increase was only found at 12 months of age, of ∼1.5-fold (Fig. 1D).

The levels of NEFL were much higher in CSF than in plasma for all the Huntington’s disease mouse lines, as well as their littermate controls, at all ages. Wild-type NEFL levels could be compared between two ages for R6/2:Q90, zQ175 and YAC128, and whilst there was a trend towards an increase with age (Fig. 1F–H), this was only statistically significant for the wild-type mice from the YAC128 colony (Fig. 1H). NEFL CSF levels were raised as compared with wild-type littermates for all of the Huntington’s disease lines at all of the ages tested. They were 3.8-fold higher for R6/2:Q200 mice at 12 weeks of age (Fig. 1E); 4-fold and 3.2-fold higher for R6/2:Q90 mice at 16 weeks and 24 weeks of age, respectively (Fig. 1F); 3.4-fold and 2.8-fold higher for zQ175 mice at 6 months and 12 months of age, respectively (Fig. 1G); and 2.4-fold and 2.8-fold higher in YAC128 mice at 6 months and 12 months of age, respectively (Fig. 1H). Therefore, elevated levels of NEFL were present in YAC128 CSF at 6 months, an age at which they had not been detected in plasma (Fig. 1D).

### Total-Tau levels were elevated only at later stage in R6/2:Q200, R6/2:Q90, zQ175 and YAC128 compared with wild-type mice

Unlike NEFL, total-Tau levels were not raised in plasma or CSF from wild-type mice at any of the ages tested (Fig. 2A–H). For the Huntington’s disease lines, elevated total-Tau levels as compared with wild-type littermate were only detected in plasma (Fig. 2A–D) and CSF (Fig. 2E–H) from mice with later-stage disease. For R6/2:Q200, there was a 5.1-fold increase at 12 weeks (Fig. 2A); for R6/2:Q90, there was a 5-fold increase at 24 weeks (Fig. 2B); for zQ175, there was a 3-fold increase at 12 months (Fig. 2C); and for YAC128, there was a 5-fold increase at 12 months of age (Fig. 2D). As for NEFL, CSF total-Tau levels were higher than plasma levels for all wild-type and Huntington’s disease mice at all ages. Total-Tau was increased in R6/2:Q200 CSF by 1.8-fold at 12 weeks (Fig. 2E), in R6/2:Q90 by 1.8-fold at 24 weeks (Fig. 2F), in zQ175 by 1.6-fold at 12 months (Fig. 2G) and in YAC128 by 1.7-fold at 12 months of age (Fig. 2H). Therefore, the relative increase in total-Tau levels was greater in plasma than in CSF.

### BRP-39 levels were elevated with disease progression in R6/2:Q200, R6/2:Q90, zQ175 and YAC128 compared with wild-type mice

BRP-39 levels increased with age in plasma from wild-type mice from each of the four colonies (Fig. 3A–D). For the R6/2:Q200 and R6/2:Q90 lines, this occurred in a stepwise manner from 2–12 weeks and 4–14 weeks, respectively (Fig. 3A and B); for the zQ175 and YAC128 lines, wild-type levels were higher at 12 months of age than at 2 months (Fig. 3C and D).

The increase in BRP-39 with age was greater for the Huntington’s disease mice than for their wild-type littermates (Fig. 3A–D). Consequently, BRP-39 was increased in R6/2:Q200 mice by 1.3-fold and 1.7-fold at 8 and 12 weeks of age, respectively (Fig. 3A); in R6/2:Q90 by 1.5-fold and 1.6-fold at 14 and 24 weeks of age, respectively (Fig. 3B); in zQ175 by 1.3-fold and 1.6-fold at 6 and 12 months of age, respectively (Fig. 3C); and in YAC128 by 1.3-fold and 1.4-fold at 6 and 12 months of age, respectively (Fig. 3D). Interestingly, in general, there was less variability in the BRP-39 plasma data sets compared with those for the NEFL and total-Tau.

### Correlations between NEFL, total-Tau and BRP-39 levels

**Figure 4:**
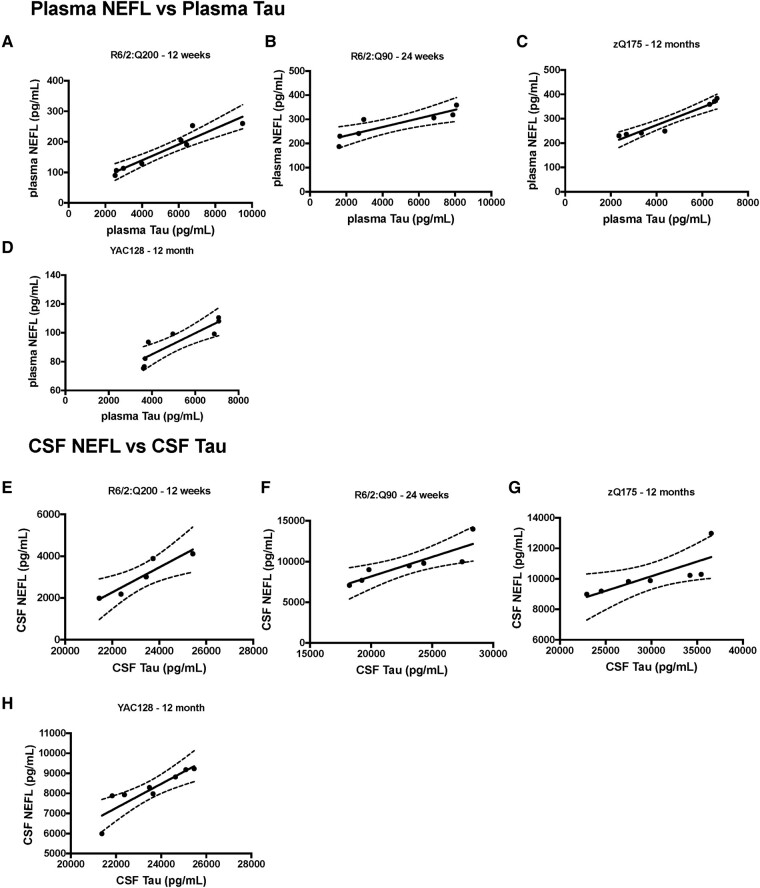
**Correlation of plasma NEFL and total-Tau and CSF NEFL and total-Tau levels at late-stage disease in R6/2:Q200, R6/2:Q90, zQ175 and YAC128 mice.** (**A**–**D**) NEFL and total-Tau plasma levels plotted for the same mice at late-stage disease for R6/2:Q200, R6/2:Q90, zQ175 and YAC128 mice. NEFL plasma levels were significantly correlated with plasma total-Tau levels in (**A**) R6/2:Q200 (*r*_P_ = 0.9565, *P* = 0.0002), (**B**) R6/2:Q90 (*r*_P_ = 0.8807, *P* = 0.0088), (**C**) zQ175 (*r*_P_ = 0.9657, *P* = 0.0004) and (**D**) YAC128 (*r*_P_ = 0.8853, *P* = 0.0035) (**E**–**H**) NEFL and total-Tau CSF levels plotted for the same mice at late-stage disease for R6/2:Q200, R6/2:Q90, zQ175 and YAC128 mice. NEFL CSF levels were significantly correlated with CSF total-Tau levels in (**E**) R6/2:Q200 (*r*_P_ = 0.9319, *P* = 0.0211), (**F**) R6/2:Q90 (*r*_P_ = 0.8639, *P* = 0.0122), (**G**) zQ175 (*r*_P_ = 0.7899, *P* = 0.0211) and (**H**) YAC128 (*r*_P_ = 0.8855, *P* = 0.0034). Data points represent a single mouse *n* = 3–5 gender/genotype. Statistical analysis was Pearson’s correlation. Curved lines represent 95% confidence bands for the linear fit. *r*_P_, Pearson’s correlation coefficient.

**Figure 5:**
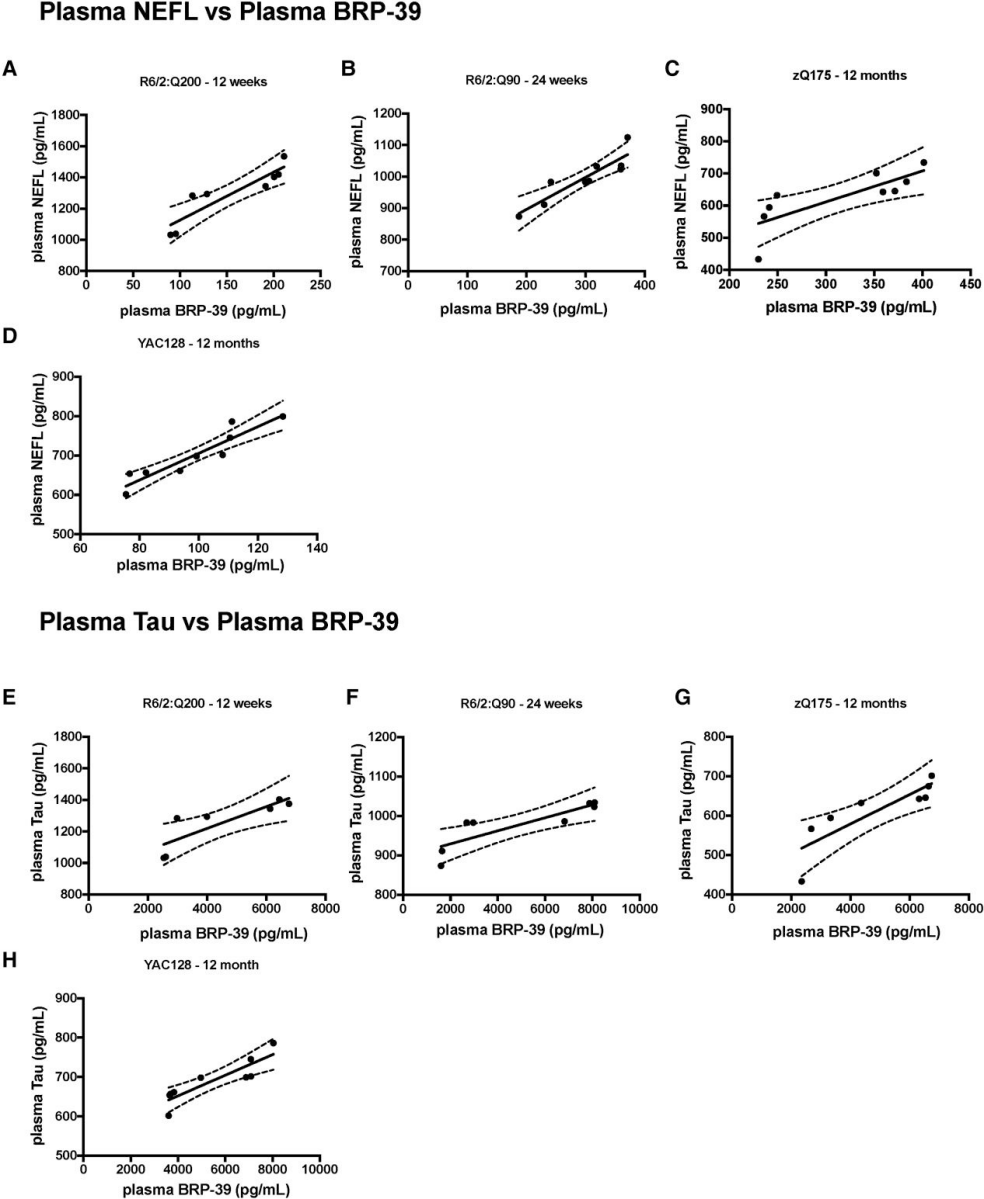
**Correlation of plasma NEFL and BRP-39 and plasma total-Tau and BRP-39 at late-stage disease in R6/2:Q200, R6/2:Q90, zQ175 and YAC128 mice.** (**A**–**D**) Plasma NEFL and BRP-39 levels plotted for the same mice at late-stage disease for R6/2:Q200, R6/2:Q90, zQ175 and YAC128 mice. NEFL plasma levels were significantly correlated with plasma BRP-39 levels in (**A**) R6/2:Q200 (*r*_P_ = 0.9056, *P* = 0.0020), (**B**) R6/2:Q90 (*r*_P_ = 0.903, *P* = 0.0008), (**C**) zQ175 (*r*_P_ = 0.7903, *P* = 0.0113) and (**D**) YAC128 (*r*_P_ = 0.9292, *P* = 0.0001). (**E**–**H**) Plasma total-Tau and BRP-39 levels plotted for the same mice at late-stage disease for R6/2:Q200, R6/2:Q90, zQ175 and YAC128 mice. Total-Tau plasma levels were significantly correlated with plasma BRP-39 levels in (**E**) R6/2:Q200 (*r*_P_ = 0.8339, *P* = 0.0391), (**F**) R6/2:Q90 (*r*_P_ = 0.8529, *P* = 0.0071), (**G**) zQ175 (*r*_P_ = 0.8513, *P* = 0.0073) and (**H**) YAC128 (*r*_P_ = 0.8809, *P* = 0.0017). Data points represent a single mouse *n* = 3–5 gender/genotype. Statistical analysis was Pearson’s correlation. Curved lines represent 95% confidence bands for the linear fit. *r*_P_, Pearson’s correlation coefficient.

We evaluated whether there was a correlation between the levels of the biomarkers in plasma and CSF in mice at the later stages of disease. For NEFL and total-Tau, we were able to examine correlations for both plasma (Fig. 4A–D) and CSF (Fig. 4E–H) and found levels to be significantly correlated in each case. We were able to compare NEFL and BRP-39 plasma levels (Fig. 5A–D) and total-Tau and BRP-39 plasma levels (Fig. 5E–H). Again, these were significantly correlated.

## Discussion

The ability to measure the impact of treatments on disease will be essential to the success of therapeutic development, and fluid biomarkers will play a critical role in the assessment of target engagement and the response to treatment. Whilst the search for novel fluid biomarkers is ongoing,^49^ neurofilament light chain has been shown to have great prognostic value,^27,28^ and total-Tau and YKL-40 have also shown to be elevated in CSF from Huntington’s disease mutation carriers.^30,31^ We set out to track these three proteins in plasma and CSF from mouse models of Huntington’s disease to provide a translatable fluid biomarkers as a readout for preclinical therapeutic trials. We chose the R6/2 transgenic, zQ175 knock-in and YAC128 transgenic mice as representative mouse models of Huntington’s disease and collected cross-sectional CSF and plasma samples at a series of ages. The R6/2:Q200 mice reach end-stage disease at 12 weeks of age and the R6/2:Q90 at 24 weeks; for these two lines, samples were collected at presymptomatic, mid-stage and late-stage disease. The zQ175 and YAC128 mice do not reach end-stage disease before 18 months and 24 months, respectively. For these two lines, samples were collected at 2, 6 and 12 months of age, chosen as they are likely to span a preclinical therapeutic trial performed in these mouse models. We developed a novel Quanterix immunoassay for BRP-39, the mouse homologue of YKL-40. NEFL, total-Tau and BRP-39 were all elevated in biofluids from Huntington’s disease mice and have the potential to provide useful biomarkers in preclinical assessments.

Neurofilament light chain is well established as a prognostic biomarker for Huntington’s disease and may have value for the monitoring of safety and therapeutic response.^27,50^ NEFL concentrations in both plasma and CSF are higher in Huntington’s disease subjects before disease onset, and baseline levels show independent prognostic power for subsequent disease onset, progression and brain atrophy.^28^ Neurofilaments are neuron-specific proteins of the axonal cytoskeleton,^51^ and NEFL is proving to be a biomarker of neuronal damage and cell death in CSF and/or plasma for a range of neurodegenerative diseases.^52,53^ We measured NEFL levels in plasma and CSF for four mouse models of Huntington’s disease and their respective wild-type littermates. In all cases, wild-type plasma levels increased with age consistent with reports for normal aging populations.^54,55^ This was true even for young mice, e.g. between 4 and 12 weeks of age for the two R6/2 colonies. There was some variation in wild-type levels between mouse colonies, the source of which is not clear. It was not related to inbred strain background; the R6/2:Q90 and YAC128 lines were both backcrossed to C56BL/6J females (Charles River), but at 6 months of age, the levels of NEFL in plasma were different. It was also not due to the batches of kits, as the levels for a given line were reproducible.

NEFL plasma and CSF levels increased with phenotype progression in all four Huntington’s disease lines and were higher in CSF than in plasma. This is consistent with previous data showing that NEFL increases in serum and CSF from R6/2 mice with ∼280 CAGs and correlated with disease severity.^32^ We detected elevated NEFL in plasma from R6/2:Q90 mice at 4 weeks of age, but not from R6/2:Q200 mice, consistent with the earlier appearance of nuclear HTT aggregation and transcriptional dysregulation in the R6/2:Q90 line.^35^ However, plasma NEFL levels do not necessarily correspond to the equivalent stages of phenotype progression between the lines. For example, at 6 months of age, plasma NEFL levels were comparable between R6/2:Q90 and zQ175 mice, an age at which R6/2:Q90 mice are close to end-stage disease,^35^ whereas although zQ175 mice have significant HTT aggregate pathology in their brains,^37^ they are yet to develop any progressive behavioural phenotypes.^36^ Plasma NEFL levels were lower in YAC128 than in zQ175 mice at comparable ages, which would be consistent with their slower onset of pathology and disease progression.^40,42^ However, CSF NEFL levels showed a different pattern; R6/2:Q90 mice at end-stage disease (24 weeks) had higher levels than zQ175 and YAC128 mice at 6 months of age, which would better reflect the stage of phenotype progression for these two lines.

Total-Tau levels have been shown to be elevated in Huntington’s disease mutation carriers as compared to healthy controls and to be associated with phenotypic variability in the disease.^30^ Tau, a microtubule-associated protein, also primarily expressed in neurons, regulates many cellular processes such as microtubule dynamics, synaptic plasticity and neurite outgrowth.^56-58^ Post-translational modifications of Tau tightly regulate these processes, and abnormal hyperphosphorylation leads to the formation of neurofibrillary tangles that interfere with fundamental cellular mechanisms.^59^ Although classically associated with Alzheimer’s disease, similar Tau dysregulations have also been reported in Huntington’s disease.^60-63^ Our analysis of total-Tau levels in plasma and CSF of the four Huntington’s disease mouse lines showed that total-Tau concentrations were only elevated at close to end-stage disease for the two R6/2 lines. For the YAC128 and zQ175 lines, increased total-Tau levels, equivalent to those found in the R6/2 lines, were detected at 12 months of age, long before these lines would reach end-stage disease. Interestingly, unlike NEFL, the fold increases in total-Tau as compared with wild type for all mouse lines were lower in CSF than in plasma. Could the expression of Tau in the enteric nervous system^64^ contribute to the plasma levels? HTT aggregates have been described in the gastrointestinal tract of mouse models of Huntington’s disease,^65^ and the loss of enteric neuropeptides has been linked to weight loss in these mice.^66^ Nevertheless, although well-established within the field of dementias, where the functions of Tau are well characterized, it remains mostly speculative in Huntington’s disease and the rate at which changes in brain pathology are reflected in plasma versus CSF total-Tau levels remains to be investigated.

In a follow-up study to a previous report,^67^ elevated levels of YKL-40 were measured in the CSF of Huntington’s mutation carriers, and the authors concluded that this protein may be a biomarker for Huntington’s disease after further investigation.^31^ YKL-40 (also known as chitinase 3-like-1 and human cartilage glycoprotein 39) and its mouse homologue, BRP-39, are chitinase-like proteins that are produced by a variety of cells including neutrophils, monocytes, macrophages, chondrocytes, synovial cells, smooth muscle cells, endothelial cells and tumour cells.^68-70^ Increased levels of YKL-40 protein have been noted in patients with a broad spectrum of pathologies and neurological disorders, but its precise function remains largely unknown.^71-76^ Unlike NEFL and Tau, which are exclusively neuronal, YKL-40 is secreted by microglia during inflammation,^77^ is expressed in astrocytes^78^ and is associated with astrocytosis and astrocytic mobility.^79^ Plasma BRP-39 increased with age in wild-type mice for each of the four colonies. The age at which increased levels in the Huntington’s disease mice could first be detected, as compared with wild-type littermates, was similar to NEFL, except BRP-39 had not increased in plasma from R6/2:Q90 mice at 4 weeks of age. Unlike NEFL, zQ175 and YAC128 mice had similar levels of BRP-39 at comparable ages.

Our work demonstrates that NEFL, total-Tau and BRP-39 all have potential as translational, preclinical fluid biomarkers in mouse models of Huntington’s disease (Supplementary Table 1) and gives an indication of the relative disease-related changes, time-courses and CSF to plasma relationships in four mouse models. Both NEFL and BRP-39 were elevated in samples from presymptomatic animals, whereas total-Tau levels were not increased in mice until later stages of disease. We found a high correlation between the levels of NEFL and total-Tau in CSF as well as all three pairwise combinations of the biomarkers in plasma. Unfortunately, we did not have sufficient CSF to validate and run the BRP-39 assays but speculate that BRP-39 is likely to be raised in the CSF of Huntington’s disease mice and to correlate with NEFL and total-Tau levels. NEFL is a marker of neuronal damage, but levels did not correlate with comparable stages of disease in these mouse models. Plasma and CSF NEFL levels were similar between R6/2:Q200 mice at 12 weeks of age and R6/2:Q90 mice at 14–16 weeks, when R6/2:Q200 mice are close to end-stage disease, but R6/2:Q90 mice will live beyond 24 weeks. Similarly, the fold increase in NEFL as compared with wild-type littermates was greater in zQ175 mice as compared with R6/2:Q90 at 6 months of age and yet end-stage disease in zQ175 is not until ∼18 months. Neither did total-Tau levels track with disease progression; elevated levels in plasma were similar for all four lines, as was also the case for CSF, despite the models being at very different phenotypic stages. Unlike NEFL, BRP-39 levels were very comparable between zQ175 and YAC128 mice, and if BRP-39 is a marker of neuroinflammation, the mechanism driving this is uncoupled from the neuronal damage that leads to increased plasma NEFL. Our plasma BRP-39 data showed less variation than NEFL and support the hypothesis that YKL-40 could be an important CSF biomarker for Huntington’s disease.^31^ The use of these fluid biomarkers in the validation of therapeutic targets and in preclinical drug screens could not only be important in the translation of therapies from mouse models to the clinic but may also shed light on the pathologies underlying these elevated biomarker levels.

## Supplementary Material

fcae030_Supplementary_Data

## Data Availability

The authors confirm that all data supporting the findings of this study are available within the article and its supplementary material. Raw data will be shared by the corresponding author on request.
