# Supplementary material for: Translatable plasma and CSF biomarkers for use in mouse models of Huntington’s disease
Source: Brain Commun. 2024 Feb 7;6(1):fcae030. doi: 10.1093/braincomms/fcae030 (PMC10873584; doi:10.1093/braincomms/fcae030)
Supplement: fcae030_Supplementary_Data [file fcae030_supplementary_data.pdf]

SUPPLEMENTARY MATERIAL

Translatable plasma and CSF biomarker for use in mouse models of Huntington disease

Supplementary Table 1. Power calculations for detecting improvements in the biomarker levels.

	**R6/2:Q200**		**R6/2:Q90**		**zQ175**		**YAC128**	
	8 wks	12 wks	14-16 wks	24 wks	6 mths	12 mths	6 mths	12 mths
NEFL CSF		70%	65%	45%		30%		25%
Total-Tau CSF		20%		40%		50%		20%
NEFL plasma	80%	80%	40%	65%	40%	45%		55%
Total-Tau plasma		65%		65%		65%		40%
BRP-39 plasma	60%	35%	30%	30%	70%	45%	50%	40%

The table indicates the percentage improvement that could be detected between Huntington's disease models and wild type mice if n = 10 with an 80% chance of detecting the improvement (p = 0.5). Power calculations were as previously described.[1] Wks = weeks, mths = months.

Supplementary Table 2. Two-way ANOVA for plasma NEFL Simoa analysis (Fig. 1)

	**R6/2:Q200 at 4, 8 and 12 weeks of age**
Genotype	F(1,54) = 14.12, p<0.0001
Age	F(2,54) = 37.82, p< 0.0001
Age x Genotype	F(2,54) = 11.74, p=0.0004
	**R6/2:Q90 at 4, 14 and 24 weeks of age**
Genotype	F(1,54) = 64.76, p<0.0001
Age	F(2,54)= 115.7, p< 0.0001
Age x Genotype	F(2,54) = 9.243, p= 0.0004
	**zQ175 at 2, 6 and 12 months of age**
Genotype	F(1,54) = 92.61, p<0.0001
Age	F(2,54) = 65.13, p<0.0001
Age x Genotype	F(2,54) = 26.57, p<0.0001
	**YAC128 at 2, 6 and 12 months of age**
Genotype	F(1,51) = 6.566, p=0.0134
Age	F(2,51) = 79.3, p<0.0001
Age x Genotype	F(2,51) = 8.415, p=0.0007

Supplementary Table 3. Two-way ANOVA or two tailed student *t*-test for CSF NEFL Simoa analysis (Fig. 1)

	**R6/2:Q200 at 12 weeks of age**
Two-tailed Student t-test	t(11)= 3.408, p = 0.0058
	**R6/2:Q90 at 14 and 24 weeks of age**
Genotype	F (1,28) = 54.93, p<0.0001
Age	F (1,28) = 32.94, p<0.0001
Age x Genotype	F(1,28) = 7.101, p=0.0126
	**zQ175 at 6 and 12 months of age**
Genotype	F(1,28) = 59.04, p<0.0001
Age	F(1,28) = 59.9, p<0.0001
Age x Genotype	F(1,28) = 11.43, p=0.0021
	**YAC128 at 6 and 12 months of age**
Genotype	F(1,28) = 104.5, p<0.0001
Age	F(1,28) = 145, p<0.0001
Age x Genotype	F(1,28) = 35.6, p<0.0001

Supplementary Table 4. Two-way ANOVA for plasma total-Tau Simoa analysis (Fig. 2)

	**R6/2:Q200 at 4, 8 and 12 weeks of age**
Genotype	F(1,42) = 21.79, p<0.0001
Age	F(2,42) = 15.82, p<0.0001
Age x Genotype	F(2,42) = 17.52, p<0.0001
	**R6/2:Q90 at 4, 14 and 24 weeks of age**
Genotype	F(1,42) = 17.15, p=0.0002
Age	F(2,42) = 6.696, p=0.0030
Age x Genotype	F(2,42) = 7.048, p=0.0023
	**zQ175 at 2, 6 and 12 months of age**
Genotype	F (1, 38) = 9.321, p=0.0041
Age	F (2, 38) = 5.203, p=0.0101
Age x Genotype	F (2, 38) = 11.2, p<0.0001
	**YAC128 at 2, 6 and 12 months of age**
Genotype	F (1, 42) = 50.03, p<0.0001
Age	F (2, 42) = 21.96, p<0.0001
Age x Genotype	F (2, 42) = 19.83, p<0.0001

Supplementary Table 5. Two-way ANOVA or two tailed student *t*-test for CSF total-Tau Simoa analysis (Fig. 2)

	**R6/2:Q200 at 12 weeks of age**
Two-tailed Student t-test	t(7)=13.26 , p <0.0001
	**R6/2:Q90 at 14 and 24 weeks of age**
Genotype	F (1, 28) = 253.3, p<0.0001
Age	F (1, 28) = 265.6, p<0.0001
Age x Genotype	F (1, 28) = 194.9, p<0.0001
	**zQ175 at 6 and 12 months of age**
Genotype	F (1, 28) = 24.5, p<0.0001
Age	F (1, 28) = 24.82, p<0.0001
Age x Genotype	F (1, 28) = 18.58, p=0.0002
	**YAC128 at 6 and 12 months of age**
Genotype	F (1, 28) = 253.3, p<0.0001
Age	F (1, 28) = 265.6, p<0.0001
Age x Genotype	F (1, 28) = 194.9, p<0.0001

Supplementary Table 6. Two-way ANOVA for plasma BRP-39 Simoa analysis (Fig. 3)

	**R6/2:Q200 at 4, 8 and 12 weeks of age**
Genotype	F (1, 30) = 52.38, p<0.0001
Age	F (2, 30) = 136.2, p<0.0001
Age x Genotype	F (2, 30) = 21.06, p<0.0001
	**R6/2:Q90 at 4, 14 and 24 weeks of age**
Genotype	F (1, 54) = 105.8, p<0.0001
Age	F (2, 54) = 320.5, p<0.0001
Age x Genotype	F (2, 54) = 27.26, p<0.0001
	**zQ175 at 2, 6 and 12 months of age**
Genotype	F (1, 54) = 48.25, p<0.0001
Age	F (2, 54) = 67.9, p<0.0001
Age x Genotype	F (2, 54) = 10.02, p=0.0002
	**YAC128 at 2, 6 and 12 months of age**
Genotype	F (1, 54) = 33.62, p<0001
Age	F (2, 54) = 287.2, p<0.0001
Age x Genotype	F (2, 54) = 23.01, p<0.0001

REFERENCES

1. Hockly E, Woodman B, Mahal A, Lewis CM, Bates G. Standardization and statistical approaches to therapeutic trials in the R6/2 mouse. *Brain research bulletin*. Sep 30 2003;61(5):469-479.
